# Stressed out symbiotes: hypotheses for the influence of abiotic stress on arbuscular mycorrhizal fungi

**DOI:** 10.1007/s00442-016-3673-7

**Published:** 2016-06-27

**Authors:** Niall S. Millar, Alison E. Bennett

**Affiliations:** 1 School of Life Sciences, University of Dundee, Dundee, DD1 4HN UK; 2 Ecological Sciences, James Hutton Institute, Errol Road, Invergowrie, Dundee, DD2 5DA UK

**Keywords:** Soil, Adaptation, Community, Symbiosis, Climate change

## Abstract

Abiotic stress is a widespread threat to both plant and soil communities. Arbuscular mycorrhizal (AM) fungi can alleviate effects of abiotic stress by improving host plant stress tolerance, but the direct effects of abiotic stress on AM fungi are less well understood. We propose two hypotheses predicting how AM fungi will respond to abiotic stress. The stress exclusion hypothesis predicts that AM fungal abundance and diversity will decrease with persistent abiotic stress. The mycorrhizal stress adaptation hypothesis predicts that AM fungi will evolve in response to abiotic stress to maintain their fitness. We conclude that abiotic stress can have effects on AM fungi independent of the effects on the host plant. AM fungal communities will change in composition in response to abiotic stress, which may mean the loss of important individual species. This could alter feedbacks to the plant community and beyond. AM fungi will adapt to abiotic stress independent of their host plant. The adaptation of AM fungi to abiotic stress should allow the maintenance of the plant-AM fungal mutualism in the face of changing climates.

**Electronic supplementary material:**

The online version of this article (doi:10.1007/s00442-016-3673-7) contains supplementary material, which is available to authorized users.

## Introduction

Abiotic stress is widespread. While abiotic stress is common in all environments, its effects are best documented in agricultural systems where abiotic stresses can cause losses in yield of food crops of up to 70 % (Mantri et al. 2011). Drought (Pardo 2010; Cramer et al. 2011), temperature (Weis and Berry 1988), salinity (Munns and Tester 2008), pH (Yokota and Ojima 1995; Koyama et al. 2001; Hinsinger et al. 2003), and nutrient deficiency or excess all negatively impact plant fitness. Arbuscular mycorrhizal (AM) fungi can often help alleviate the negative consequences of these stresses.

The arbuscular mycorrhizal symbiosis is an important relationship formed between the members of the phylum Glomeromycota and ~ 80 % of all land plants (Smith and Read 2008). AM fungi are obligate symbionts that colonise plant roots. The fungi gain carbohydrates from the plant host, while the fungi improve plant nutrient and water uptake. The benefits to plant partners can vary depending on the AM fungal species, plant species, and abiotic context (Hoeksema et al. 2010). AM fungal community composition and diversity are influenced by plant community composition and diversity (Johnson et al. 2004; Hausmann and Hawkes 2009; De Deyn et al. 2011; Koch et al. 2012; López-García et al. 2014; Chagnon et al. 2015; Reininger et al. 2015), biotic stress (Eom et al. 2001; Gehring and Bennett 2009), and abiotic factors (Johnson et al. 1992; Zobel and Öpik 2014; Antoninka et al. 2015; Borriello et al. 2015; Klabi et al. 2015). In addition, these factors often interact to influence AM fungal community structure and diversity (e.g., Johnson et al. 1992; Klabi et al. 2015). Despite our awareness of the influence of these factors on AM fungal community structure and diversity, predominantly only the evolutionary influence of plant community composition on AM fungi has been explored (Kiers and Van Der Heijden 2006; Wyatt et al. 2014; but see Behm and Kiers 2014; Johnson 1993).

The AM symbiosis has been shown to reduce the negative effects of abiotic stresses. In this study, we define abiotic stress as a shift in any non-living factor within the environment away from the optimal condition or away from the condition to which most organisms in that environment have become adapted. While abiotic stress is context dependent, there are a number of examples demonstrating the impact of AM fungi in improving abiotic stress tolerance in plants. AM fungi improve plant fitness during drought (Smith and Read 2008) possibly due to the increased surface area for water absorption provided by AM hyphae (Auge 2001), increased access to small soil pores (Smith and Read 2008), or improved apoplastic water flow (Bárzana et al. 2012). Improved phosphorus nutrition is a common benefit of the AM symbiosis, and particularly during drought conditions, AM fungi improve P uptake from dry soil (Neumann and George 2004). AM fungal-improved salinity tolerance (Al-Karaki 2000; Evelin et al. 2009) has been hypothesised to be due to improved P nutrition, improved ion homeostasis, maintaining photochemical capacity, and higher activity of antioxidant enzymes (Hajiboland et al. 2009). Heavy metal toxicity for plants can be reduced by AM fungi, through hyphal ‘metal binding’ which reduces the bioavailability of elements, such as Cu, Pb, Co, Cd, and Zn (Audet and Charest 2007). AM fungi may also be more tolerant than plant roots of high temperatures (Bunn et al. 2009), and induce higher enzymatic activity and secondary metabolite content in plants (Chen et al. 2013) leading to greater cold tolerance in host plants. As a result, AM fungi can clearly benefit host plants exposed to abiotic stress.

As mentioned above, less attention has been paid to the direct selective effects of abiotic stress on AM fungi themselves, independent from the effect on their host. Abiotic stress also impacts host plants, and therefore, abiotic stress will indirectly influence AM fungi via host plants, although this influence is likely to follow patterns similar to those identified for the influence of plants on AM fungi under ambient conditions (Kiers and Van Der Heijden 2006; Wyatt et al. 2014). As a result, in this study, we address the possible direct effects of abiotic stress on the fitness, diversity, evolution, community composition, and symbiotic functioning of AM fungi. Studying the effects of abiotic stress on AM fungi separately from plants will help to provide a better understanding of the strengths and weaknesses of their ubiquitous relationship.

Like in all organisms, environmental variation causes selection for different traits in AM fungi. This leads to individuals differing in their symbiotic function based on the contrasting climates or soil conditions of the areas they originated from (Mena-Violante et al. 2006; Antunes et al. 2011; Sochacki et al. 2013). It has been suggested that local adaptation to varying environmental conditions produce more important differences in AM fungi than the basic functional differences between coexisting AM fungal taxa (Sanders 2002). Adaptation to environmental conditions can even be seen in highly localised areas, for example, within a natural CO_2_ spring, where hypoxia has driven the selection of AM fungal species capable of surviving high concentrations of CO_2_ resulting in AM fungi with reduced extra-radical mycelia and enhanced uptake of oxygen from the roots of the plant (Maček et al. 2011). Similarly, the ability of three AM fungal phylotypes from Yellowstone National Park to survive in geothermal soils is likely due to tolerance of low pH conditions (Appoloni et al. 2008). Evolutionary responses to abiotic stress not only improve the ability of an AM fungus to survive, but may also benefit the host plants exposed to the same stress (Mena-Violante et al. 2006; Sochacki et al. 2013). This may not be equally true for all stresses, however. For example, increased nutrient loads have been suggested to reduce the benefit AM fungi deliver to host plants (Johnson 1993; Antunes et al. 2012). Only in extreme cases are nutrients present in such excess as to directly damage plants (Scheirs and De Bruyn 2004), but many changes in nutrient availability away from the level plants and fungi are adapted to can be considered an abiotic stress, and, in addition, nutrient availability can undermine the benefits that plants receive from the mycorrhizal symbiosis (Johnson 1993). The potential for AM fungi to adapt to novel conditions may be a particularly important characteristic for an organism with limited dispersal capabilities.

The benefits provided to plants by AM fungi will become even more important, due to increasing abiotic stresses caused by climate change (Hanson and Weltzin 2000; Compant et al. 2010). This is demonstrated by the knock-on effects on plant communities that can occur when AM fungal diversity or community composition is changed (van der Heijden et al. 1998; Antoninka et al. 2009; Sun et al. 2013). Knock-on effects occur if plants differ in their response to specific AM fungal species, or if they have varying levels of general dependence on the symbiosis. In this case, a change in AM fungal community composition could, for example, make a species of plant dependent on the AM symbiosis (or a particular AM fungal species) less fit. It could then be out-competed by plant species less dependent on AM fungi (Mariotte et al. 2013). Given the potential influence of AM fungi on plant responses to climate change, the direct effects of abiotic stress on the fungi themselves and the consequences for the symbiosis cannot be ignored.

In this study, we present two hypotheses for how abiotic stress influences the ecology and evolution of AM fungi (Table 1). The stress exclusion hypothesis addresses the ecological consequences for an AM fungal community of a relatively short-term abiotic stress, whereas the stress adaptation hypothesis examines the long-term evolutionary consequences of selection by abiotic stress on AM fungi.

**Table 1:** Summary of the stress exclusion and mycorrhizal stress adaptation hypotheses including assumptions, possible experiments to test assumptions currently lacking evidence, and predictions

Hypothesis	Assumptions	Possible experiments	Predictions
Stress exclusion: abiotic stress will cause changes in AMF community composition.	1. AMF communities are diverse		1. Applying an abiotic stress to a soil with a diverse AMF community will change the AMF community composition. Species that cannot tolerate adverse conditions will be the first to become less prevalent, leaving the soil system with a higher proportion of species tolerant to that stress. This will result in an overall change in composition in the AMF community
	2. Abiotic stresses affect AMF directly		
	3. AMF species can differ in their response to the same abiotic stress, some having a negative reaction that reduces their abundance		
	4. Abiotic stress response of AMF is not dependent on/controlled by host plant response		
Mycorrhizal stress adaptation: abiotic stress will lead to adaption among AMF species within communities from areas that are repeatedly exposed to abiotic stress.	1. AMF and plants are equally likely to interact under ambient or abiotic stress conditions		1. Abiotic stress adapted AMF will have greater nutrient delivery function under the specific abiotic stress to which they have adapted than AMF that have not adapted to the same abiotic stress 2. Host plant fitness will improve under abiotic stress conditions in the presence of AMF adapted to that abiotic stress condition versus no AMF 3. Host plant fitness will improve under abiotic stress conditions in the presence of AMF adapted to that abiotic stress condition versus AMF that are not adapted to that abiotic stress condition
	2. AMF benefit host plants under abiotic stress		
	3. AMF adapt directly to abiotic stress conditions		
	4. Plant adaptations to abiotic stress conditions do not influence AMF adaptation to abiotic stress	4. Grow two genotypes of a plant species with varying tolerance to an abiotic stress with AMF under the abiotic stress or ambient conditions. After multiple generations, inoculate unadapted plant genotype, grow with and without the stress, and compare AM fungal fitness	
	5. AMF adaptation to abiotic stress conditions improves AMF fitness		
	6. AMF adaptation to abiotic stress conditions will influence different plant species and communities equally		
	7. AMF adaptation to one abiotic stress will not result in adaptation to all abiotic stresses	7. Inoculate plants with AMF and grow under one abiotic stress. After multiple generations, inoculate plants with selected AMF, grow under different abiotic stresses, and compare AMF fitness	

*AMF* arbuscular mycorrhizal fungi

## The stress exclusion hypothesis

**Fig. 1:**
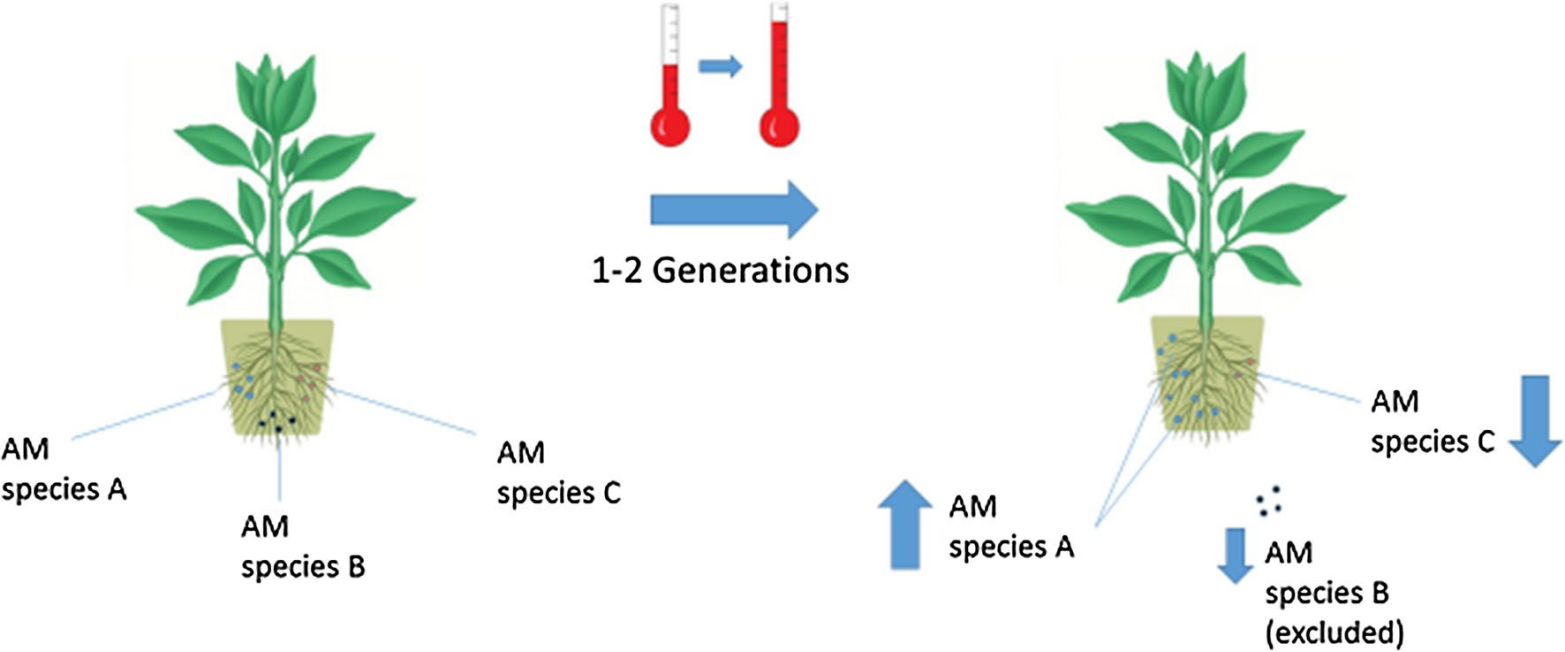
Representation of the prediction of the stress exclusion hypothesis. Thermometers showing a higher temperature represent a more intense abiotic stress, and thermometers showing a lower temperature represent a less intense abiotic stress. The predictions of the stress exclusion hypothesis suggest that since different AM fungal species have varying responses to abiotic stress, species with a poorer tolerance to abiotic stress (such as species B in this figure) will be reduced in abundance or even excluded from the AM fungal community

We hypothesise that abiotic stress will reduce the diversity of AM fungi (Fig. 1). We know that abiotic stress directly affects plant fitness, and abiotic stress can also directly affect the fitness of AM fungi (Fitter et al. 2000; Staddon et al. 2004). AM fungal species vary greatly in functional (Munkvold et al. 2004; Powell et al. 2009), phenotypic, and genetic diversity (Klironomos et al. 2001; Treseder and Allen 2002; Antunes et al. 2011), and thus, we expect that an abiotic stress will affect individual AM fungal species differently, leading to variation in AM fungal fitness. As a result, abiotic stress in this system can act as an environmental filtering process (Vellend 2010; HilleRisLambers et al. 2012). Specifically, depending on the severity and duration of the stress, less-tolerant AM fungal species may be entirely excluded from the community. Thus, we expect abiotic stress to reduce AM fungal diversity and alter AM fungal community composition resulting in an AM fungal community with a higher proportion of species that are more phenotypically similar, because they are more tolerant of that specific abiotic stress.

### Assumptions

We make four main assumptions in this hypothesis (Table 1):

*First, we assume that AM fungal communities are diverse* The diversity of undisturbed AM fungal communities is well established. Currently, there are around 240 known species in the phylum Glomeromycota (Redecker et al. 2013), but it is likely that there are many more unidentified species (Ohsowski et al. 2014). AM fungal communities contain many species, and single plants may associate with as many as 20 different fungi (Fitter 2005).

*Second, we assume that abiotic stresses can affect AM fungi* There are several examples where abiotic stress has been shown to directly influence AM fungi. Extra-radical hyphae (ERH) respond directly to temperature and moisture (Allen and Kitajima 2013), and variation in temperature experienced by ERH can also influence intra-radical colonisation by AM fungi (Heinemeyer and Fitter 2004) and nutrient transfer to and growth of host plants (Barrett et al. 2014). However, prolonged warming can cause a reduction in respiration in the extra-radical mycelia (Heinemeyer et al. 2006). By contrast, exposure to winter-like conditions (4 °C) for periods as short as 2 weeks have been shown to cause abnormal germination in *Glomus intraradices* spores (Juge et al. 2002). These spores grew short, recurved hyphae that are associated with stressful conditions (Rayner and Coates 1987). Extended cold storage (>90 days) maximised proper germination with longer ranged, straight hyphae (Juge et al. 2002). Freezing temperatures also have a negative effect on the extent of colonisation by AM fungi in plant roots (Klironomos et al. 2001). Drought has been shown to have both positive and negative effects on AM fungal colonisation of host plants (Auge 2001; Klironomos et al. 2001), but in some cases, drought has caused a reduction in ERH or even led to the inability of AM fungi to colonise plant roots and, therefore, reduced the fungal fitness (Compant et al. 2010; Neumann et al. 2010). On the other end of the scale, flooding has been shown to reduce AM fungal diversity (Deepika and Kothamasi 2014). Heavy metal pollution has been shown to specifically affect AM fungal ERH by inhibiting their growth (Del Val et al. 1999). Thus, there is extensive evidence that AM fungi are directly affected by abiotic stress (reviewed in Lenoir et al. 2016).

*Third, not all AM fungal species respond to abiotic stress in the same way* Not all plants respond to abiotic stress equally, so we should not be surprised that not all AM fungi respond to abiotic stress equally. AM fungi are functionally diverse (Munkvold et al. 2004; Powell et al. 2009), and vary both phenotypically and genetically in response to abiotic stress (Klironomos et al. 2001; Treseder and Allen 2002; Antunes et al. 2011), suggesting that abiotic stress likely influences each fungal species differently. This can be linked to differences in AM fungal communities between climatic zones. For example, communities from mesic sites are characterised by the presence of Gigasporaceae and Acaulosporaceae, while communities from semiarid sites are characterised by Glomeraceae and Paraglomaceae (Egerton-Warburton et al. 2007). *Rhizophagus irregularis* and *Claroideoglomus etunicatum* increased hyphal and arbuscular colonisation of host plant roots in response to elevated CO_2_ levels, while *Acaulospora denticulata* and *Scutellospora calospora* showed no response to elevated CO_2_ (Klironomos et al. 1998). Two other *Glomus* species also showed increased density of ERH in response to elevated CO_2_ (Staddon et al. 2004). Warm conditions promote higher root colonisation rates by *Ambispora leptoticha* than in either *Claroideoglomus claroideum* or *Funneliformis mosseae*, while under cold conditions, this pattern is reversed (Antunes et al. 2011). The response of AM fungi to freezing temperatures shows variation both between and within genera, with three *Glomus* species showing much greater tolerance to exposure to −5 °C for 4 weeks than either *S. calospora* or *A. denticulata* (Klironomos et al. 2001). Conversely, *S. calospora* and *A. denticulata* showed increased root colonisation in response to drought, while the root colonisation of the same three *Glomus* species was reduced (Klironomos et al. 2001). AM fungal species have also been shown to differ in their response to soil fertility and fertilisation. For example, *Glomus* species are more abundant in N-fertilised soil, whereas *Scutellospora* species are more abundant in P-fertilised soil (Treseder and Allen 2002). P limitation appears to promote mutualistic phenotypes in AM fungi, while N limitation promotes commensal or even parasitic phenotypes (Johnson et al. 2014). *C. claroideum* has been shown to have a higher tolerance of heavy metal toxicity than *F. mosseae.C. claroideum* does not show the inhibition in ERH growth that other *Glomus* species do (Del Val et al. 1999). There is thus significant evidence for variation between AM fungi for response to abiotic stress.

AM fungal responses to abiotic stress can also be influenced by phenotypic plasticity within AM fungal species (Behm and Kiers 2014). Phenotypic plasticity would be an added advantage for AM fungal species exposed to frequent, but transient, abiotic stress, because it would allow fungi to maintain fitness but not lose genetic variation useful in the absence of the stress. As a result, those AM fungal species that persist in the face of an abiotic stress may be more plastic than those that do not.

*Fourth, we assume that the response of AM fungi to abiotic stress is not controlled by or entirely dependent on the response of the host plant* AM fungi are obligate symbionts, and they depend on their hosts for carbohydrates. Given this close relationship, the response of the plant to the abiotic stress cannot be ignored, but it does not override the direct response of the fungi themselves. The ERH and spores of the fungi are exposed directly to the soil conditions. Experiments involving compartmentalised ERH (Heinemeyer and Fitter 2004; Heinemeyer et al. 2006; Barrett et al. 2014) show that AM fungi can respond directly to stresses applied to their ERH without any response from the plant.

### Prediction

With these assumptions in mind, we make the following prediction.

Applying an abiotic stress to a soil system with a diverse AM fungal community will reduce the fitness of certain species of AM fungi, causing them to decrease in abundance or be excluded from the community. This will result in a compositional change in the community.

We expect that some AM fungal species have a poorer tolerance to a given abiotic stress. For example, *Glomus* spp. has a lower root colonisation under phosphorus fertilisation than *S. calospora* possibly due to an intolerance of low root carbohydrate concentrations (Pearson et al. 1994). Drought stress halved arbuscule and vesicle formation in *Glomus fasciculatum* and reduced total root colonisation by a third as compared with *Glomus* strain ZAC-19. Warming has been shown to have a greater effect on rare species of AM fungi than more common species in the community, causing a significant reduction in their abundance (Sun et al. 2013) and possibly local extinction for those AM fungal species. Nitrogen and phosphorus fertilisation can significantly reduce the number of operational taxonomic units within an AM fungal community and shift community composition (Camenzind et al. 2014). Communities fertilised with N show a reduced richness of Diversisporales, while communities fertilised with P show a reduced richness of Glomerales while combined N and P fertilisation lead to losses of rare species (Camenzind et al. 2014). Similarly, chronic N deposition has been shown to significantly reduce the amount of rare AM fungal taxa in a community (van Diepen et al. 2013). Short-term periods of drought can have a stimulatory effect on root colonisation and sporulation of AM fungi, but the lower spore production and species richness of AM fungal communities from arid areas indicate the long-term negative impacts of drought (Auge 2001). Thus, there is evidence from multiple studies of abiotic stress influences on AM fungi to support our prediction (Supplementary Table 1).

At this stage, adaptive phenotypic plasticity may be responsible for the ability of certain species to tolerate an abiotic stress, such as can be seen in the response of *F. mosseae* to varying levels of moisture and heat (Stahl and Christensen 1991). Whether or not the tolerant phenotype persists if the abiotic stress abates will test the potential for reversibility in phenotypic plasticity (Behm and Kiers 2014). Conversely, the reduction of certain species in the community could be due to non-adaptive phenotypic plasticity producing phenotypes that are less able to tolerate the abiotic stress (Ghalambor et al. 2007). Either way, we expect that overall fungal abundance and/or species richness will likely decrease.

One potential modifier of the compositional changes that an abiotic stress will cause could be the dispersal of AM fungal propagules from other locations. AM fungi can be spread to new locations by birds (Nielsen et al. 2016), mammals (Fracchia et al. 2011), and possibly wind in drier habitats (Egan et al. 2014). It appears that the AM fungal species best suited to dispersal produce more spores and have higher colonisation potentials, but also are less competitive and are not as persistent (Nielsen et al. 2016). This suggests that compositional changes caused by abiotic stress are unlikely to be reversed by dispersal. Environmental variables appear to be more important than dispersal in structuring mycorrhizal communities (Lekberg et al. 2007). However, a particularly extreme, but transient, abiotic stress (such as a flood, or volcanic activity) could provide the circumstances where dispersal would be more important than adaptation for the reestablishment of the AM fungal community. In this case, early successional species may have more of an advantage than stress-tolerant species, but this will be a rare exception rather than the rule.

## Mycorrhizal stress adaptation hypothesis

**Fig. 2:**
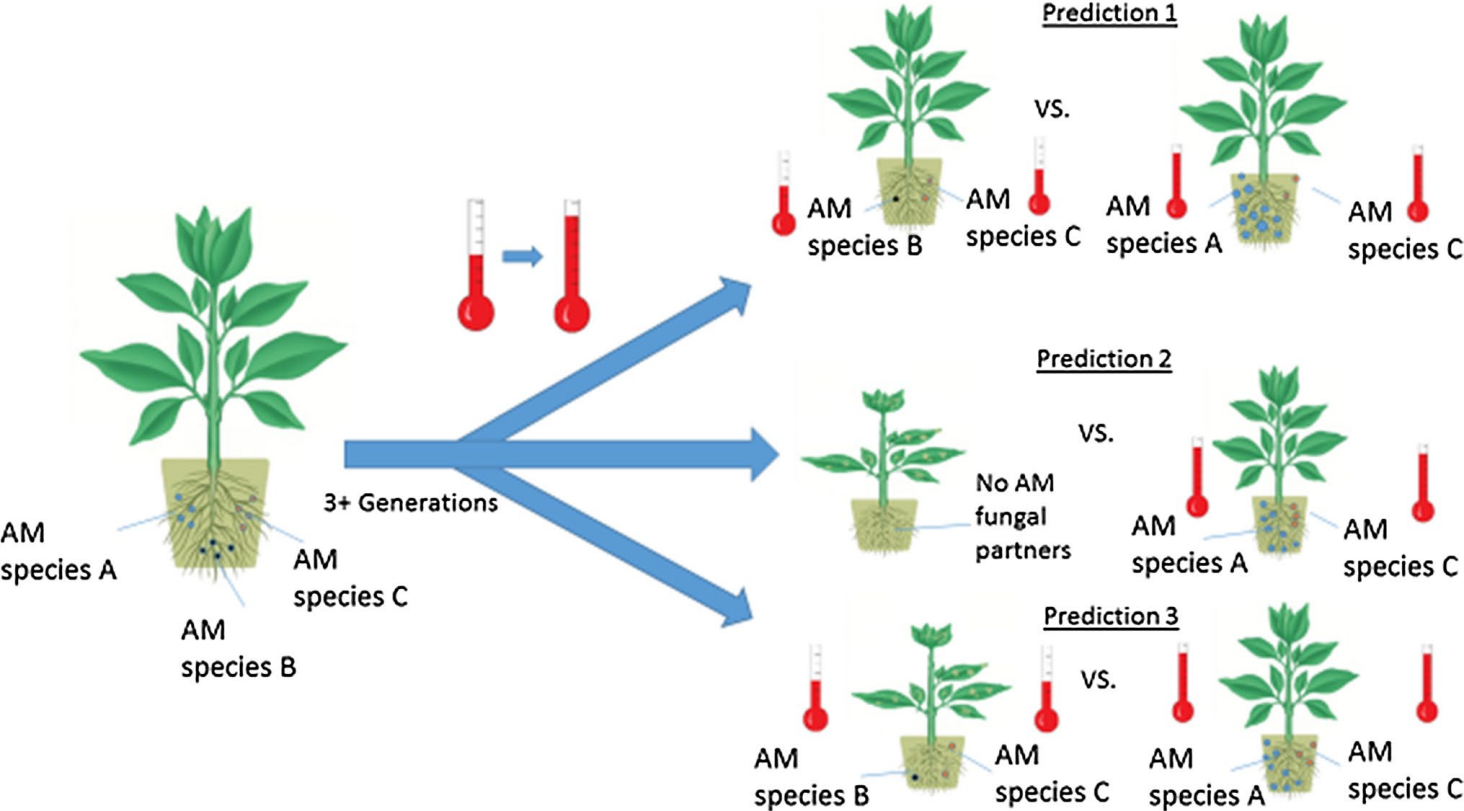
Representation of the three predictions of the mycorrhizal stress adaptation hypothesis using high temperature as an example abiotic stress. Selection has led to phenotypic changes within three generations in AM fungi in the previous studies (Angelard et al. 2014), and so three generations was used as the cutoff to represent changes due to selection in AM fungi. In the first prediction, stress-adapted AM fungal species are expected to maintain a higher level of fitness when exposed to an abiotic stress than unadapted species [as represented by increases in spore number (a frequent proxy for AM fungal fitness) in the figure]. In the second prediction, plants hosting stress-adapted AM fungal partners are expected to be more tolerant of that stress than plants hosting no AM fungi. Finally, in the third prediction, plants hosting stress-adapted AM fungal partners are expected to be more tolerant to that stress than plants hosting unadapted AM fungal partners. The lack of tolerance in host plants is reflected by stunted growth and leaf spots in this figure

After an abiotic stress has caused the reduction or exclusion of AM fungal species that have a poorer tolerance to that stress, the community will be left with a higher proportion of species that are better able to tolerate that given stress. If the stress is not transient, then the surviving AM fungal species exposed to abiotic stress will undergo selection and adaptation to that stress. Adaptation to an abiotic stress should improve the fitness of an AM fungal species (Fig. 2). This means that AM fungal communities from areas that repeatedly or continuously experience abiotic stress factors such as drought, salinity, heavy metal pollution, nutrient deposition, and extremes of temperature will contain species that are adapted specifically to their environment and may benefit their hosts more under that abiotic stress condition than non-adapted AM fungal ecotypes (Appoloni et al. 2008; Maček et al. 2011).

### Assumptions

We make seven assumptions in this hypothesis (Table 1):

*First, we assume that AM fungi and plants are equally likely to interact under ambient or abiotic stress conditions* The association between AM fungi and plants under abiotic stress is well documented. For example, the AM symbiosis is present in well-watered and drought conditions (Morte et al. 2000; Porcel and Ruiz-Lozano 2004; Bárzana et al. 2012), and at high and low temperatures (Klironomos et al. 2001; Bunn et al. 2009). Total root colonisation may not remain constant during abiotic stress, but even relatively low levels of colonisation can support a mutualistic symbiosis (Sochacki et al. 2013). The degree of association may change due to abiotic stress; however, there is significant evidence to suggest that association (regardless of degree) will be maintained.

*Second, we assume that AM fungi continue to conduct resource exchange with their host plants when they are exposed to an abiotic stress* Here, we assume that during an abiotic stress, AM fungi will also continue to supply their host with nutrients. That is, they will maintain symbiotic function in addition to colonising their host plants.

There is a wealth of information on the ability of AM fungi to benefit plants during abiotic stress conditions (Smith and Read 2008), including drought (Aroca and Ruiz-Lozano 2009; Auge 2001), nutrient deficiency (Smith and Read 2008), and salinity (Evelin et al. 2009). An abiotic stress, however, can reduce the benefit provided to plants by AM fungi (such as nutrient excess caused by fertilisation (Johnson 1993) or shading that reduces potential carbon available for allocation to AM fungi (Zheng et al. 2015). This is particularly likely if the cost of supporting an AM fungal partner exceeds the benefit provided by the symbiosis (Johnson 1993, 2010; Johnson et al. 1997; Neuhauser and Fargione 2004), and may lead to reduced allocation to AM fungi by a plant partner (Zheng et al. 2015).

We know of no direct evidence for this assumption, but one could test the assumption in the following way: inoculate plants with a common AM fungal inoculum, and subject half of the plants to an abiotic stress, while the other half are grown under ambient conditions. Allow the plants to grow for multiple generations before isolating the AM fungi from the soil, and using them to inoculate new plants exposed to the abiotic stress or ambient conditions. Add radioactively labelled phosphorus to pots and compare the phosphorus uptake between plants in both conditions to determine whether exposure to abiotic stress has compromised the nutrient delivery function of the AM fungal inocula. If this assumption is true, then nutrient uptake should still occur in plants inoculated with AM fungi previously exposed to abiotic stress.

*Third, we assume that AM fungi can respond directly to the effects of abiotic stress* Specifically, we assume that abiotic stress will cause selection within AM fungal species, for improved stress tolerance. This can be seen in the response of AM fungi to long-term nutrient deficiency (Johnson et al. 2010; Antunes et al. 2012), drought (Davies et al. 2002), and heavy metal pollution (Del Val et al. 1999). There is a range of mechanisms that AM fungi have developed to deal with abiotic stresses like these. Drought tolerance can be achieved by improved water uptake and protection from protein denaturation (Porcel et al. 2006; Querejeta et al. 2006). Heat shock can be mitigated in a similar fashion (Ocón et al. 2007). Salt tolerance has been hypothesised to result from upregulation of genes encoding chaperones or aquaporins (Estrada et al. 2013). Stress from pollutants like heavy metals can be mitigated by preventing oxidative damage (Lanfranco et al. 2005), and degradation (Song et al. 2016), sequestration (González-Chávez et al. 2004), or transportation (Gonzalez-Guerrero et al. 2005) of the pollutants themselves. Thus, abiotic stress appears to select for adapted AM fungal ecotypes.

*Fourth, we assume that the host plants’ own evolutionary response will not be more important for maintaining the fitness of an AM fungal species than adaptation by an AM fungal species itself* If the abiotic stress in question is present over the course of multiple plant generations, then it is likely that the plants will exhibit adaptations of their own (Hirt and Shinozaki 2004). Currently, there is evidence of the beneficial effects that the adaptations of a fungal symbiont can have on their host plants (Rodriguez et al. 2010; Redman et al. 2011; Southworth 2012). However, it is not known what, if any, effect the host plant’s own adaptations have on a fungal partner, plant adaptation to abiotic stress will likely be important to the AM fungi, but we expect AM fungal adaptation to be equally (or more) important for maintaining AM fungal fitness under abiotic stress.

Given that there is no direct evidence for this assumption, we propose the following experiment: grow two genotypes of a plant species, varying in adaptation to a particular abiotic stress with a common AM fungal inoculum under abiotic stress or ambient conditions. After multiple generations of plant growth, both selected and unselected AM fungi should be isolated and used to inoculate plants of the unadapted plant genotype and grown with and without the stress. Comparing the fitness of the AM fungi in these conditions will reveal any effects that adaptation by the host plant had on the AM fungi. If this assumption is true, then there should be little difference in the fitness of the AM fungi with and without the abiotic stress. We expect that this experiment will show that AM fungi cannot rely entirely on the adaptations of their host plant, and that adaptations of their own are needed to improve their fitness.

Host plant species are clearly important in structuring AM fungal communities (Eom et al. 2000), but abiotic soil conditions are equally important (Johnson et al. 1992). Vice versa, AM fungal communities can be important in structuring the community of their hosts (van der Heijden et al. 1998). The genetically variable nuclei that AM fungi have give them the potential to respond rapidly to changes in their host plant and their environment (Angelard et al. 2014). In a changing environment, soil microbes may have a greater capacity to adapt than their hosts, given their shorter generation time (Lau and Lennon 2012). This shows that AM fungi, despite being obligate symbionts, can adapt independent of any one particular host plant species, and should not be considered as entirely dependent passengers in the symbiosis.

*Fifth, we assume that the adaptation of an AM fungus to an abiotic stress will improve the fitness of that AM fungus* An improvement in fitness will be indicated by an increase in the combined parameters of percent root colonisation, extra-radical hyphal length, and spore number. The best evidence for this is nutrient excess which selects for AM fungal ecotypes with a higher fitness (often measured by a combination of root colonisation and extra-radical hyphae), although these ecotypes are also frequently less mutualistic (Johnson 1993; Johnson et al. 1997; Neuhauser and Fargione 2004). As a result, there is evidence for adaptations to abiotic stress increasing AM fungal fitness.

*Sixth, we assume that the adaptations gained by an AM fungal species will improve their fitness and maintain host plant associations independent of the specific host plant species they were associated with when initially exposed to the abiotic stress* When an AM fungal species is first exposed to an abiotic stress, it will be associated with a host plant, but we assume that regardless of the initial host species, an AM fungus was associated with during a stress and that AM fungal species will still associate with multiple plant species within a community. When exposed to an abiotic stress, preadapted AM fungi may provide plants with better tolerance. For example, AM fungi isolated from arid soils promoted pepper growth under drought (Mena-Violante et al. 2006). A study on the effects of AM fungi from 42 different soils on a novel host plant, *Lotus corniculatus* L., showed that adaptation to the edaphic factors of an AM fungal site of origin was more important than host identity for promoting a mutualistic relationship (Lambert et al. 1980). Thus, AM fungal adaptation to an abiotic stress will not be dependent upon the host plant, and it was in association with when the abiotic stress was applied.

*Seventh, we assume that adaptation to a particular abiotic stress in AM fungi will not result in adaptation to all abiotic stresses* Both plants and AM fungi are likely to be exposed to a wide variety of abiotic stresses, but the traits that confer tolerance to a stress in either organism are not likely to provide tolerance to all stresses. Tradeoffs in adaptation to different abiotic stresses have long been documented in all organisms, and therefore, we should expect there to be tradeoffs in adaptation to different abiotic stresses in AM fungi. For example, adaptation to nitrogen and water availability has been shown to appear in different AM fungal strains, but not the same strain (Martinez-Garcia et al. 2015). While some adaptations, such as enhanced osmotic adjustment in roots (Porcel and Ruiz-Lozano 2004), could be useful both during low-water and high-salinity stresses, specific adaptations will not be applicable to all possible abiotic stresses. The same will likely be true for AM fungi.

There are relatively few tests in the literature applicable to this assumption (but see Martinez-Garcia et al. 2015), so we propose the following experiment to test this assumption. Inoculate a number of plants with the same AM fungi and grow them under one abiotic stress with all other conditions being non-limiting. For example, plants and AM fungi grown under drought stress should also be grown with optimal temperature, salinity, soil chemistry, and nutrients. After multiple generations of plant growth, isolate the AM fungi from the soil and use as inocula for a new set of plants. Grow replicated inoculated plants under different abiotic stresses. Comparing the fitness of the AM fungi using a metric incorporating spore abundance, intra- and extra-radical hyphal growth, should show whether exposure to one abiotic stress has improved AM fungal tolerance to other abiotic stresses. If this assumption is true, then the fitness of the AM fungi under novel abiotic stresses should be lower than that under the initial abiotic stress. Adaptation to a specific abiotic stress will improve the fitness of an AM fungus, but only under the initial abiotic stress.

### Predictions

With these assumptions in mind, we make three main predictions in this hypothesis.

*First, we predict that AM fungi that are adapted to a particular abiotic stress will have greater fitness when exposed to that stress than AM fungi that have not previously been exposed to that stress* We expect adaptation to an abiotic stress should promote AM fungal fitness. Again, changes in fitness would be best measured using a metric combining spore abundance, intra- and extra-radical hyphal growth, as AM fungal species are represented at varying levels between these measures (Varela-Cervero et al. 2015). For example, previous research suggests that drought-adapted AM fungi may have spores with a higher drought tolerance (Jacobson 1997) which would increase their fitness when exposed to drought. Adaptation to an abiotic stress will, therefore, improve AM fungal fitness.

The strength of selection can be limited by dispersal (reviewed in Räsänen and Hendry 2008), particularly in the case of microbes (Hanson et al. 2012). AM fungal dispersal is typically limited to belowground hyphal development (Smith and Read 2008), but recent studies have shown that AM fungal spores can be dispersed by wind in arid environments (Egan et al. 2014), rodents (Mangan and Adler 2002; Fracchia et al. 2011), and potentially by birds (Nielsen et al. 2016). However, the rate of dispersal by these mechanisms is low (Egan et al. 2014), suggesting that in many environments, dispersal is unlikely to greatly limit selection. However, in the case of extreme but transient stresses, this low level of dispersal could aid in restoring genetic diversity when an abiotic stress was absent.

While adaptive phenotypic plasticity may have been responsible for a species’ survival of an abiotic stress, this does not preclude the possibility for adaptive evolution as well. An adaptive evolutionary response is likely to follow beneficial phenotypic plasticity if the new phenotype is not yet optimal, and because exclusion (or extinction) is made less likely (Ghalambor et al. 2007).

*Second, we predict that plants associated with AM fungi preadapted to an abiotic stress will improve under that abiotic stress as compared to plants grown without any AM fungal partners* Association with AM fungi is generally more beneficial to plants under abiotic stress (Smith and Read 2008), and preadapted AM fungi have been shown to benefit host plants. For example, AM fungi preadapted to drought improve drought tolerance in plants as compared with plants grown without AM fungi (Marulanda et al. 2007; Sochacki et al. 2013), even when plants not associated with AM fungi were supplied with extra phosphorus (Davies et al. 2002). As a result, we expect stress-adapted AM fungi to benefit host plants (Supplementary Table 1).

*Third, we predict that fitness of plants associated with prestress*-*adapted AM fungi will improve when exposed to that abiotic stress as compared to plants associated with non*-*adapted AM fungi* If adaptation by AM fungi improves fungal fitness, we might expect that benefit to also transfer to host plants, especially in comparison with plants associated with AM fungi that have not undergone adaptation in response to the focal stress. For example, AM fungi from well-watered areas confer less drought tolerance for host plants than AM fungi from drought prone regions (Davies et al. 2002; Martinez-Garcia et al. 2015). AM fungi adapted to serpentine soils have been shown to improve plant growth and phosphorus uptake in serpentine soils as compared with AM fungi from other soils (Doubková et al. 2012). AM fungal adaptations, therefore, likely maintain the fitness of both plants and AM fungi, and these adaptations are likely to become more important with climate changes. The widespread occurrence of abiotic stresses are already a big problem for agriculture (van Velthuizen et al. 2007; Mantri et al. 2011), and thus, AM fungal adaptations could help maintain worldwide food security.

## Implications

Natural plant communities are subjected to abiotic stresses that are associated with their environment, as are their associated AM fungi, and it is important to consider how the symbiosis will affect, and be effected by, these pressures, especially if AM fungi that are adapted to abiotic stress may help alleviate the effects of abiotic stress in host plants.

### Plant-fungal community feedbacks

Some plants are more responsive to particular AM fungal species than others (Hartnett and Wilson 1999), so reductions in the abundance of key AM fungal species will in turn reduce the abundance of certain plant species. Dependence on a single AM fungal species within a community may become more common if species identity is important in providing tolerance to an extreme abiotic stress (Rodriguez and Redman 2008; Zabinski and Bunn 2014). It could also be possible for the loss of a plant species to feed back to AM fungal species leading to losses of other important AM fungi from communities. Thus, the loss of one partner in the mycorrhizal symbiosis has the potential to cause the loss of other partner species. High levels of co-dependence in plants and AM fungi will be particularly important during abiotic stress, as the loss or reduction of either partner may intensify the negative effects of an abiotic stress.

AM fungi can alter competition between plant species (Mariotte et al. 2013; Lin et al. 2015). The level of mycorrhizal dependence of plant species in a community can influence how AM fungi alter competitive interactions (Urcelay and Díaz 2003). The right AM fungi can improve the ability of competing plant species to coexist (Klabi et al. 2014), so a reduction in AM fungal abundance or diversity could alter competitive outcomes between plants leading to a change in plant community composition and diversity (Cahill et al. 2008). Plant species that are less dependent on the mycorrhizal symbiosis will have a competitive advantage when AM fungal abundance is reduced (Scheublin et al. 2007), allowing them to become more dominant in the community. Thus, abiotic stress may both directly impact plant and AM fungal communities, but may also indirectly impact both communities via altered feedbacks between partners.

### AM fungal adaptation influences on plants

AM fungal adaptations to abiotic stress will improve AM fungal fitness, and these adaptations could have variable effects on the host plant. Adaptations by AM fungal species to abiotic stress have been hypothesised to have positive (e.g., Martinez-Garcia et al. 2015; Mena-Violante et al. 2006) or negative (e.g., Johnson 1993; Neuhauser and Fargione 2004) consequences for the maintenance of the mutualistic symbiosis (Kiers and van der Heijden 2006; Johnson et al. 1997). Under abiotic stress, nutrient exchange may not be the only mycorrhizal function that influences plant fitness. An AM fungal species may also be considered mutualistic if it provides tolerance to abiotic stress. Abiotic stresses that affect plants by means other than limiting their access to nutrients could promote a mutualistic relationship based on factors other than resource exchange. For example, AM fungi adapted to sites with high concentrations of heavy metals may alleviate plant toxicity by preventing heavy metals from accessing sensitive areas in plant roots, or by excreting metal chelators (Schützendübel and Polle 2002; Miransari 2011; Seguel et al. 2013). Thus, adaptations by AM fungi could result in novel benefits for host plants.

However, changes in AM fungal community composition associated with abiotic stress could alter the functional composition of AM fungal communities as well (Finlay 2008; Feddermann et al. 2010). AM fungi have been hypothesised, like plants, to fall into three life-history categories: competitors, ruderals, and stress-tolerant species (Chagnon et al. 2013). We can imagine that if an ambient community begins with equal proportions of AM fungi of each type of life-history, that an abiotic stress, in accordance with the stress exclusion hypothesis, is likely to shift the proportion of functional types strongly in favour of stress-tolerant species. In this case, the community may lose more ruderal species, or those best able to colonise new hosts in an environment or disperse to new patches, and competitive species. Thus, abiotic stress could limit the range of functions (particularly types of associations with host plants) as well as the abundance of functions within an AM fungal community.

### Placing abiotic stress in the broader context of selective forces on AM fungi

While AM fungal adaptations are likely to influence plants, the reverse, which host plant plays an important role in structuring AM fungal communities (e.g., Eom et al. 2000; Johnson et al. 2004), is also true. Host plant specificity for AM fungal species has partially been credited for this role; however, plants can also affect the abiotic properties of their soil (e.g., Bezemer et al. 2006). As a result, plants could indirectly alter their AM fungal community by changing soil abiotic properties. In addition, many of the adaptations to abiotic stress of plants will also alter soil abiotic properties which could directly or indirectly impact AM fungi. In particular, in very low nutrient environments, plants exude organic acids to release bound nutrients (Lambers et al. 2015), and plants can also increase water availability near the soil surface by growing deep tap roots. As a result, AM fungal associations with plants are likely partially due to specificity and partially due to the abiotic environment created by a host plant.

In addition, to the selective pressures created by abiotic stress and plants, AM fungi also face biotic stress pressures. Very little is known about the direct influence of biotic stress on AM fungal adaptation, despite the presence of fungal grazers, such as Collembola and nematodes. Greater information is available on the influence on AM fungi of biotic stresses on their host plants. Plant herbivory has a range of effects on AM fungi including both increasing and reducing root colonisation, and reduction in species diversity (Eom et al. 2001; Gehring and Bennett 2009). Adaptations by AM fungi that improve their host plant’s defence response may increase both partners’ fitness, but this has rarely been explored in an evolutionary context (but see Bennett et al. 2006; Rasmann et al. 2011). As a result, there is scope for increasing our understanding of the importance of abiotic stress in relation to plant host and biotic stress for driving selection on AM fungi.

### Climate change

With the advance of climate change, plants will be exposed to more extreme abiotic stresses (Fitzpatrick et al. 2008; Lindner et al. 2010; Benito et al. 2014). Increased abiotic stress will likely affect the geographic ranges of plants, and could increase the impact on AM fungi due to their reduced ability to migrate in response to a changing environment (Fitter et al. 2000). The diversity and abundance of AM fungi and plants are correlated, so we expect that a change in one partner will lead to a change in diversity or abundance of the other partner. This correlation will be particularly important given the potential for AM fungi to mediate the response of plants to climate change (Mohan et al. 2014), so any changes in the AM fungal community, particularly if they lead to changes in AM fungal function, are sure to have repercussions for plants. Even under ambient conditions, changes in the composition of an AM fungal community can affect the composition of a plant community (van der Heijden et al. 1998; Pellissier et al. 2013). Climate changes will likely alter the diversity of both plants and AM fungi and the dominant species in both communities could change. If abiotic stress caused by climate changes reduces AM fungal abundance, it is likely that non-mycotrophic plants will benefit more than mycotrophic plants, as some non-mycotrophic plants, such as *Salsola kali*, can be suppressed by AM fungi (Antoninka et al. 2009). The presence of non-mycotrophic plants can make it difficult for arid sites to be recolonised by mycotrophic plants. For example, plants like garlic mustard can suppress AM fungi in the soil and make it harder for mycotrophic plants to associate with them (Roberts and Anderson 2001; Koch et al. 2011; Lankau et al. 2014). Unlike non-mycotrophic plants, mycotrophic plants may require AM fungi to survive abiotic stress conditions (Allen and Allen 1988; Olsson and Tyler 2004; Lambers et al. 2015). In this way, climate changes could cause a sequence of positive feedbacks that further reduce AM fungal abundance and diversity and increase impacts on plant communities.

With the potential for both the expansion and reduction in ranges of certain plants, a shift in dominance toward C_4_ plants, and changes in AM fungal community composition, it is clear that climate changes will significantly alter natural plant and soil systems. Climate changes could lead to an increased abundance of C_4_ plants due to their greater ability to take advantage of elevated levels of CO_2_ (Bloom et al. 2012; Morgan et al. 2011; Pendall et al. 2011). This could have a positive effect on AM fungal abundance, because C_4_ plants have been shown to be more responsive to AM fungi (Hetrick et al. 1990; Bennett et al. 2013). In addition to higher responses to AM fungi, elevated levels of CO_2_ can also stimulate AM fungal colonisation in C_4_ more than in C_3_ plants (Monz et al. 1994). Elevated atmospheric CO_2_, warming, and decreased precipitation are inter-related consequences of climate changes, but they do not all affect AM fungi and plants in the same way. Thus, we cannot accurately predict effects of climate changes, but it is possible that elevated CO_2_ levels could promote C_4_ plants and AM fungi (Morgan et al. 2011).

The loss of important AM fungal species could have particularly strong consequences for agricultural systems in the face of climate change. Agricultural soil generally has AM fungal communities with low diversity due to intensive farming techniques (Alguacil et al. 2008; Verbruggen and Kiers 2010). Climate change is likely to intensify existing abiotic stresses and broaden the geographical range over which they affect both agricultural and natural soil systems (Lane and Jarvis 2007; Allen et al. 2010). This means that we can expect agricultural systems to be exposed to abiotic stresses like heat and drought more frequently, particularly in tropical regions (Mendelsohn and Dinar 1999). Reductions in AM fungal diversity and abundance with intensive farming practices may limit the evolutionary potential of AM fungi and the ability of the AM fungi in agricultural soil to adapt to abiotic stresses.

### Invasive species

While climate change can reduce the range of some plant species, the range of other species is increasing, particularly invasive species (Dukes and Mooney 1999; Bradley et al. 2009; Diez et al. 2012; Vicente et al. 2013). We cannot make precise predictions about how an AM fungal community would react to an invasive species in the context of abiotic stress, but it is likely that further compositional changes would be induced. Reductions in diversity of AM fungi in response to invasive species have been observed previously (Hawkes et al. 2006; Mummey and Rillig 2006; Vogelsang and Bever 2009; Shannon et al. 2014). The influence of abiotic stress on invasive species will depend on the prevailing conditions of the area they originated from, and their ability to rapidly adapt to a new environment that may also be changing. Invasive species have been noted for their ability to adapt to new environments (Prentis et al. 2008), but it is hard to predict how they interact with AM fungi. Many invasive plants do not form mycorrhizas (Pringle et al. 2009), while some do and are assisted by them (Marler et al. 1999; Reinhart and Callaway 2006; Nuñez et al. 2009). However, the reduction in density and diversity of AM fungi often associated with plant invasions (Mummey and Rillig 2006; Vogelsang and Bever 2009) could limit the ability of AM fungi in those systems to adapt to abiotic stresses. Any reduction in diversity or abundance of AM fungi will reduce the genetic variation within the AM fungal community, and this will limit their evolutionary potential (England et al. 2003). In addition, any reduction in the abundance, diversity, and evolutionary potential of AM fungi will have consequences for the survival of both above- and belowground communities, especially during an abiotic stress.

### Possible utilisation of adapted AM fungi

Adaptation to a particular abiotic stress could be taken advantage of, for example, in creating commercial AM fungal inocula. Adaptation by AM fungi creates opportunities for suppliers to produce inocula that improve the stress tolerance of a crop. Inoculating field soils with AM fungi has been shown to be effective for improving the yield of various crops under relatively benign conditions (Bagyaraj and Manjunath 1980; Pellegrino et al. 2011; Ortas 2012; Ceballos et al. 2013) and under abiotic stress (Gholamhoseini et al. 2013). Creating an AM fungal inoculum that is tailored to an abiotic stress could be a sustainable strategy to help farmers in regions where agriculture is restricted by an abiotic stress. Similarly, restoration efforts could be aided by not only inoculating with an AM fungal inoculate prior to reintroduction to the field (Richter and Stutz 2002), but by inoculating with AM fungi with abiotic stress tolerance needed for the restored environment. Ecotypes such as these could be used as an alternative to ecotypes from AM fungal banks that do not control the abiotic stresses from the source of their inocula. Another avenue in restoration using stress-adapted AM fungi could be in the phytoremediation of soils polluted by heavy metals like Cu, Zn, Pb, Co, and Cd. This involves the use of metal-accumulating plants to reduce the level of heavy metals in soils contaminated by nearby mines or the overuse of sewage sludge (Suchkova et al. 2010). This is cheaper and more environmentally friendly than other methods like the use of chemicals (Chen et al. 2000). Using AM fungi that have adapted to heavy metal toxicity can further improve phytoremediation by helping the plants survive, for example, Cu or Zn pollution (Orłowska et al. 2005; Meier et al. 2011, 2015).

While mycorrhizae are ubiquitous in nature, there are opportunities for their addition to both agricultural and natural systems, particularly when the AM fungal community of an area is degraded or unadapted.

### Stress-adapted AM fungi in non-stressed conditions

What happens to AM fungal fitness when a long-term abiotic stress ends? An example could be when long-term droughts, as in Southern California, end. When an AM fungus becomes well adapted to an abiotic stress, it will likely become a dominant ecotype in the community. In the hypoxic soils found in CO_2_ springs, two AM fungal phylotypes were found exclusively in hypoxic soil and were significantly more abundant in the community than other phylotypes found outside the CO_2_ springs (Maček et al. 2011). If the CO_2_ springs were to disappear from that site would these adapted ecotypes lose their competitive advantage? We predict these “adapted” ecotypes would likely be reduced in abundance in the community as other ecotypes perhaps better suited to the “new” environment outcompete them. If the stress periodically returns, they may still persist in the community (Fitter et al. 2000), but perhaps not as abundant as before the loss of the stress. AM fungi have demonstrated a remarkable ability to adapt to extreme environments, but such a degree of adaptation could diminish their ability to survive outside those environments.

## Conclusions

We conclude that AM fungi are important for improving plant tolerance to abiotic stress, but also respond to abiotic stress independently of their host plant. Abiotic stresses affect the abundance and community composition of AM fungi. Changes in the diversity of AM fungi will feed back into the plant community and cause corresponding changes in diversity and dominant plant species, and these feedbacks will become stronger with climate changes, agriculture, and plant invasions. AM fungi are capable of adapting to the abiotic environment which may or may not improve their mutualistic function. The impact of the ecological and evolutionary responses of AM fungi to abiotic stresses is likely to become even more important for both natural and agricultural systems in the face of climate changes and biotic stresses, such as invasion by non-native species.

## Electronic supplementary material

Below is the link to the electronic supplementary material.

Supplementary material 1 (PDF 75 kb)
