# Supplementary material for: Stressed out symbiotes: hypotheses for the influence of abiotic stress on arbuscular mycorrhizal fungi
Source: Oecologia. 2016 Jun 27;182(3):625–41. doi: 10.1007/s00442-016-3673-7 (PMC5043000; doi:10.1007/s00442-016-3673-7)
Supplement: Supplementary file 1 — Supplementary material 1 (PDF 75 kb) [file 442_2016_3673_MOESM1_ESM.docx]

| **Hypothesis** | **Assumptions** | **Predictions** | **Possible Experiments** |
| --- | --- | --- | --- |
| **The Stress Exclusion Hypothesis:**  Abiotic stress will cause changes in AMF community composition | **1)** AMF communities are diverse (Fitter 2005; Redecker et al. 2013; Ohsowski et al. 2014) **2)** Abiotic stresses affect AMF directly (Rayner and Coates 1987; Del Val et al. 1999; Klironomos et al. 2001; Juge et al. 2002; Heinemeyer and Fitter 2004; Compant et al. 2010; Neumann et al. 2010; Allen and Kitajima 2013; Deepika and Kothamasi 2014) **3)** AMF species can differ in their response to the same abiotic stress, some having a negative reaction that reduces their abundance (Klironomos et al. 1998; Klironomos et al. 2001; Treseder and Allen 2002; Staddon et al. 2004; Egerton-Warburton et al. 2007; Antunes et al. 2011; Behm and Kiers 2014) **4)** The response of AMF to abiotic stress is not dependent on or controlled by the host plant's response (Heinemeyer and Fitter 2004; Heinemeyer et al. 2006; Barrett et al. 2014) | **1)** Applying an abiotic stress to a soil with a diverse AMF community will change the AMF community composition. Species that cannot tolerate adverse conditions will be the first to become less prevalent, leaving the soil system with a higher proportion of species tolerant to that stress. This will result in an overall change in composition in the AMF community (Stahl and Christensen 1991; Pearson et al. 1994; Auge 2001; Ghalambor et al. 2007; van Diepen et al. 2013; Camenzind et al. 2014) |  |
| **Mycorrhizal Stress Adaptation:**  Abiotic stress will lead to adaption among AMF species within communities from areas that are repeatedly exposed to abiotic stress | **1)** AMF and plants are equally likely to interact under ambient or abiotic stress conditions (Morte et al. 2000; Klironomos et al. 2001; Porcel and Ruiz-Lozano 2004; Bunn et al. 2009; Bárzana et al. 2012; Sochacki et al. 2013) **2)** AMF benefit host plants under abiotic stress (Auge 2001; Smith and Read 2008; Aroca and Ruiz-Lozano 2009; Evelin et al. 2009) **3)** AMF adapt directly to abiotic stress conditions (Del Val et al. 1999; Davies et al. 2002; González-Chávez et al. 2004; Gonzalez-Guerrero et al. 2005; Lanfranco et al. 2005; Porcel et al. 2006; Querejeta et al. 2006; Ocón et al. 2007; Johnson et al. 2010; Antunes et al. 2012; Estrada et al. 2013; Song et al. 2016) **4)** Plant adaptations to abiotic stress conditions do not influence AMF adaptation to abiotic stress (Johnson et al. 1992; Lau and Lennon 2012; Angelard et al. 2014) **5)** AMF adaptation to abiotic stress conditions improves AMF fitness (Johnson 1993; Jacobson 1997; Johnson et al. 1997; Neuhauser and Fargione 2004) **6)** AMF adaptation to abiotic stress conditions will influence different plant species and communities equally (Lambert et al. 1980; Mena-Violante et al. 2006)  **7)** AMF adaptation to one abiotic stress will not result in adaptation to all abiotic stresses (Martinez-Garcia et al. 2015) | **1)** Abiotic stress adapted AMF will have greater nutrient delivery function under the specific abiotic stress to which they have adapted than AMF that have not adapted to the same abiotic stress (Ghalambor et al. 2007; Sochacki et al. 2013) **2)** Host plant fitness will improve under abiotic stress conditions in the presence of AMF adapted to that abiotic stress condition versus no AMF (Davies et al. 2002; Marulanda et al. 2007; Smith and Read 2008; Sochacki et al. 2013) **3)** Host plant fitness will improve under abiotic stress conditions in the presence of AMF adapted to that abiotic stress condition versus AMF that are not adapted to that abiotic stress condition (Davies et al. 2002; Doubková et al. 2012; Martinez-Garcia et al. 2015) | **4)** Grow two genotypes of a plant species with varying tolerance to an abiotic stress with AMF under the abiotic stress or ambient conditions. After multiple generations, inoculate un-adapted plant genotype, grow with and without the stress, and compare AM fungal fitness **7)** Inoculate plants with AMF and grow under one abiotic stress. After multiple generations, inoculate plants with selected AMF, grow under different abiotic stresses, and compare AMF fitness |

Allen MF, Kitajima K (2013) In situ high-frequency observations of mycorrhizas. New Phytol 200:222–228. doi: 10.1111/nph.12363

Angelard C, Tanner CJ, Fontanillas P, et al (2014) Rapid genotypic change and plasticity in arbuscular mycorrhizal fungi is caused by a host shift and enhanced by segregation. ISME J 8:284–94. doi: 10.1038/ismej.2013.154

Antunes PM, Koch AM, Morton JB, et al (2011) Evidence for functional divergence in arbuscular mycorrhizal fungi from contrasting climatic origins. New Phytol 189:507–514. doi: 10.1111/j.1469-8137.2010.03480.x

Antunes PM, Lehmann A, Hart MM, et al (2012) Long-term effects of soil nutrient deficiency on arbuscular mycorrhizal communities. Funct Ecol 26:532–540. doi: 10.1111/j.1365-2435.2011.01953.x

Aroca R, Ruiz-Lozano JM (2009) Induction of Plant Tolerance to Semi-arid Environments by Beneficial Soil Microorganisms – A Review. In: Climate Change, Intercropping, Pest Control and Beneficial Microrganisms, Sustainble Agriculture reviews, vol. 2. Springer Science+Business Media, pp 121–135

Auge RM (2001) Water relations, drought and vesicular-arbuscular mycorrhizal symbiosis. Mycorrhiza 11:3–42. doi: 10.1007/s005720100097

Barrett G, Campbell CD, Hodge A (2014) The direct response of the external mycelium of arbuscular mycorrhizal fungi to temperature and the implications for nutrient transfer. Soil Biol Biochem 78:109–117.

Bárzana G, Aroca R, Paz JA, et al (2012) Arbuscular mycorrhizal symbiosis increases relative apoplastic water flow in roots of the host plant under both well-watered and drought stress conditions. Ann Bot 109:1009–1017. doi: 10.1093/aob/mcs007

Behm JE, Kiers ET (2014) A phenotypic plasticity framework for assessing intraspecific variation in arbuscular mycorrhizal fungal traits. J Ecol 102:315–327. doi: 10.1111/1365-2745.12194

Bunn R, Lekberg Y, Zabinski C (2009) Arbuscular mycorrhizal fungi ameliorate temperature stress in thermophilic plants. Ecology 90:1378–1388. doi: 10.1890/07-2080.1

Camenzind T, Hempel S, Homeier J, et al (2014) Nitrogen and phosphorus additions impact arbuscular mycorrhizal abundance and molecular diversity in a tropical montane forest. Glob Chang Biol 20:3646–3659. doi: 10.1111/gcb.12618

Compant S, Van Der Heijden MGA, Sessitsch A (2010) Climate change effects on beneficial plant–microorganism interactions. FEMS Microbiol Ecol 73:197–214. doi: 10.1111/j.1574-6941.2010.00900.x

Davies FT (Jr), Olalde-Portugal V, Aguilera-Gomez L, et al (2002) Alleviation of drought stress of Chile ancho pepper (*Capsicum annuum* L. cv. San Luis) with arbuscular mycorrhiza indigenous to Mexico. Sci Hortic (Amsterdam) 92:347–359. doi: 10.1016/S0304-4238(01)00293-X

Deepika S, Kothamasi D (2014) Soil moisture–a regulator of arbuscular mycorrhizal fungal community assembly and symbiotic phosphorus uptake. Mycorrhiza 25:67–75. doi: 10.1007/s00572-014-0596-1

Del Val C, Barea JM, Azcón-Aguilar C (1999) Assessing the tolerance to heavy metals of arbuscular mycorrhizal fungi isolated from sewage sludge-contaminated soils. Appl Soil Ecol 11:261–269. doi: 10.1016/S0929-1393(98)00153-X

Doubková P, Suda J, Sudová R (2012) The symbiosis with arbuscular mycorrhizal fungi contributes to plant tolerance to serpentine edaphic stress. Soil Biol Biochem 44:56–64. doi: 10.1016/j.soilbio.2011.09.011

Egerton-Warburton LM, Johnson NC, Allen EB (2007) Mycorrhizal Community Dynamics following Nitrogen Fertilization : A Cross-Site Test in Five Grasslands. Ecol Monogr 77:527–544.

Estrada B, Barea JM, Aroca R, Ruiz-Lozano JM (2013) A native *Glomus intraradices* strain from a Mediterranean saline area exhibits salt tolerance and enhanced symbiotic efficiency with maize plants under salt stress conditions. Plant Soil 366:333–349. doi: 10.1007/s11104-012-1409-y

Evelin H, Kapoor R, Giri B (2009) Arbuscular mycorrhizal fungi in alleviation of salt stress: A review. Ann Bot 104:1263–1280. doi: 10.1093/aob/mcp251

Fitter AH (2005) Darkness visible: Reflections on underground ecology. J Ecol 93:231–243. doi: 10.1111/j.1365-2745.2005.00990.x

Ghalambor CK, McKay JK, Carroll SP, Reznick DN (2007) Adaptive versus non-adaptive phenotypic plasticity and the potential for contemporary adaptation in new environments. Funct Ecol 21:394–407. doi: 10.1111/j.1365-2435.2007.01283.x

González-Chávez MC, Carrillo-González R, Wright SF, Nichols KA (2004) The role of glomalin, a protein produced by arbuscular mycorrhizal fungi, in sequestering potentially toxic elements. Environ Pollut 130:317–323. doi: 10.1016/j.envpol.2004.01.004

Gonzalez-Guerrero M, Azcon-Aguilar C, Mooney M, et al (2005) Characterization of a *Glomus intraradices* gene encoding a putative Zn transporter of the cation diffusion facilitator family. Fungal Genet Biol 42:130–140. doi: 10.1016/j.fgb.2004.10.007

Heinemeyer A, Fitter AH (2004) Impact of temperature on the arbuscular mycorrhizal (AM) symbiosis: Growth responses of the host plant and its AM fungal partner. J Exp Bot 55:525–534. doi: 10.1093/jxb/erh049

Heinemeyer A, Ineson P, Ostle N, Fitter AH (2006) Respiration of the external mycelium in the arbuscular mycorrhizal symbiosis shows strong dependence on recent photosynthates and acclimation to temperature. New Phytol 171:159–170. doi: 10.1111/j.1469-8137.2006.01730.x

Jacobson KM (1997) Moisture and substrate stability determine VA-mycorrhizal fungal community distribution and structure in an arid grassland. J Arid Environ 35:59–75.

Johnson NC (1993) Can Fertilization of Soil Select Less Mutualistic Mycorrhizae? Ecol Appl 3:749–757.

Johnson NC, Graham JH, Smith FA (1997) Functioning of mycorrhizal associations along the mutualism–parasitism continuum. New Phytol 135:575–585. doi: 10.1046/j.1469-8137.1997.00729.x

Johnson NC, Tilman D, Wedin D (1992) Plant and soil controls on mycorrhizal fungal communities. Ecology 73:2034–2042.

Johnson NC, Wilson GWT, Bowker M a, et al (2010) Resource limitation is a driver of local adaptation in mycorrhizal symbioses. Proc Natl Acad Sci U S A 107:2093–8. doi: 10.1073/pnas.0906710107

Juge C, Samson J, Bastien C, et al (2002) Breaking dormancy in spores of the arbuscular mycorrhizal fungus *Glomus intraradices*: A critical cold-storage period. Mycorrhiza 12:37–42. doi: 10.1007/s00572-001-0151-8

Klironomos JN, Hart MM, Gurney JE, Moutoglis P (2001) Interspecific differences in the tolerance of arbuscular mycorrhizal fungi to freezing and drying. Can J Bot 79:1161–1166. doi: 10.1139/cjb-79-10-1161

Klironomos JN, Ursic M, Rillig M, Allen MF (1998) Interspecific differences in the response of arbuscular mycorrhizal fungi to *Artemisia tridentata* grown under elevated atmospheric CO_2_. New Phytol 138:599–605.

Lambert DH, Cole H, Baker DE (1980) Adaptation of vesicular-arbuscular mycorrhizae to edaphic factors. New Phytol 85:513–520.

Lanfranco L, Novero M, Bonfante P (2005) The mycorrhizal fungus *Gigaspora margarita* possesses a CuZn superoxide dismutase that is up-regulated during symbiosis with legume hosts. Plant Physiol 137:1319–30. doi: 10.1104/pp.104.050435

Lau JA, Lennon JT (2012) Rapid responses of soil microorganisms improve plant fitness in novel environments. Proc Natl Acad Sci U S A 109:14058–14062. doi: 10.1073/pnas.1202319109

Martinez-Garcia L., Manrique E, Pugnaire FI (2015) Different mycorrhizal fungal strains determine plant community response to nitrogen and water availability. J Plant Nutr 146–154.

Marulanda A, Porcel R, Barea JM, Azcón R (2007) Drought tolerance and antioxidant activities in lavender plants colonized by native drought-tolerant or drought-sensitive *Glomus* species. Microb Ecol 54:543–552. doi: 10.1007/s00248-007-9237-y

Mena-Violante HG, Ocampo-Jiménez O, Dendooven L, et al (2006) Arbuscular mycorrhizal fungi enhance fruit growth and quality of chile ancho (*Capsicum annuum* L. cv San Luis) plants exposed to drought. Mycorrhiza 16:261–267. doi: 10.1007/s00572-006-0043-z

Morte A, Lovisolo C, Schubert A (2000) Effect of drought stress on growth and water relations of the mycorrhizal association *Helianthemum almeriense-Terfezia claveryi*. Mycorrhiza 10:115–119. doi: 10.1007/s005720000066

Neuhauser C, Fargione JE (2004) A mutualism–parasitism continuum model and its application to plant–mycorrhizae interactions. Ecol Modell 177:337–352. doi: 10.1016/j.ecolmodel.2004.02.010

Neumann E, Schmid B, Römheld V, George E (2010) Extraradical development and contribution to plant performance of an arbuscular mycorrhizal symbiosis exposed to complete or partial rootzone drying. Mycorrhiza 20:13–23. doi: 10.1007/s00572-009-0259-9

Ocón A, Hampp R, Requena N (2007) Trehalose turnover during abiotic stress in arbuscular mycorrhizal fungi. New Phytol 174:879–891. doi: 10.1111/j.1469-8137.2007.02048.x

Ohsowski BM, Zaitsoff PD, Öpik M, Hart MM (2014) Where the wild things are: Looking for uncultured Glomeromycota. New Phytol 204:171–179. doi: 10.1111/nph.12894

Pearson JN, Abbott LK, Jasper DA (1994) Phosphorus, soluble carbohydrates and the competition between two arbuscular mycorrhizal fungi colonizing subterranean clover. New Phytol 127:101–106. doi: 10.1111/j.1469-8137.1994.tb04263.x

Porcel R, Aroca R, Cano C, et al (2006) Identification of a gene from the arbuscular mycorrhizal fungus *Glomus intraradices* encoding for a 14-3-3 protein that is up-regulated by drought stress during the AM symbiosis. Microb Ecol 52:575–582. doi: 10.1007/s00248-006-9015-2

Porcel R, Ruiz-Lozano JM (2004) Arbuscular mycorrhizal influence on leaf water potential, solute accumulation, and oxidative stress in soybean plants subjected to drought stress. J Exp Bot 55:1743–1750. doi: 10.1093/jxb/erh188

Querejeta JI, Allen MF, Caravaca F, Roldán a (2006) Differential modulation of host plant delta13C and delta18O by native and nonnative arbuscular mycorrhizal fungi in a semiarid environment. New Phytol 169:379–387. doi: 10.1111/j.1469-8137.2005.01599.x

Rayner ADM, Coates D (1987) Regulation of mycelial organisation and responses. In: Symposium of the British Mycological Society. Cambridge University Press,

Redecker D, Schüßler A, Stockinger H, et al (2013) An evidence-based consensus for the classification of arbuscular mycorrhizal fungi (Glomeromycota). Mycorrhiza 23:515–531. doi: 10.1007/s00572-013-0486-y

Smith SE, Read DJ (2008) Mycorrhizal Symbiosis, 3rd edn. Academic Press, New York

Sochacki P, Ward JR, Cruzan MB (2013) Consequences of Mycorrhizal Colonization for *Piriqueta* Morphotypes under Drought Stress. Int J Plant Sci 174:65–73. doi: 10.1086/668224

Song F, Li J, Fan X, et al (2016) Transcriptome analysis of *Glomus mosseae*/*Medicago sativa* mycorrhiza on atrazine stress. Sci Rep. doi: 10.1038/srep20245.

Staddon PL, Gregersen R, Jakobsen I (2004) The response of two *Glomus* mycorrhizal fungi and a fine endophyte to elevated atmospheric CO_2_, soil warming and drought. Glob Chang Biol 10:1909–1921. doi: 10.1111/j.1365-2486.2004.00861.x

Stahl PD, Christensen M (1991) Population variation in the mycorrhizal fungus *Glomus mosseae*: breadth of environmental tolerance. Mycol Res 95:300–307. doi: 10.1016/S0953-7562(09)81238-0

Treseder KK, Allen MF (2002) Direct nitrogen and phosphorus limitation of arbuscular mycorrhizal fungi: a model and field test. New Phytol 155:507–515. doi: 10.1046/j.1469-8137.2002.00470.x

van Diepen LTA, Entwistle EM, Zak DR (2013) Chronic nitrogen deposition and the composition of active arbuscular mycorrhizal fungi. Appl Soil Ecol 72:62–68. doi: 10.1016/j.apsoil.2013.05.012
